# Characterizing mJOA-defined post-surgical recovery patterns in patients with degenerative cervical myelopathy

**DOI:** 10.1016/j.wnsx.2023.100267

**Published:** 2023-12-09

**Authors:** Alexander C. Friesen, Sarah A. Detombe, Pat Doyle-Pettypiece, Wai Ng, Kevin Gurr, Chris Bailey, Parham Rasoulinejad, Fawaz Siddiqi, Robert Bartha, Neil Duggal

**Affiliations:** a Schulich School of Medicine & Dentistry, Western University, London, Ontario, Canada; b Department of Clinical Neurological Sciences, London Health Sciences Centre, London, Ontario, Canada; c Department of Orthopedics, London Health Sciences Centre, London, Ontario, Canada; d Centre for Functional and Metabolic Mapping, Robarts Research Institute, Western University, London, Ontario, Canada; e Department of Medical Biophysics, Schulich School of Medicine and Dentistry, Western University, London, Ontario, Canada

**Keywords:** Degenerative cervical myelopathy, mJOA, Neurological recovery

## Abstract

**Background:**

Degenerative cervical myelopathy is a spinal disorder resulting in progressive cord compression and neurological deficits that are assessed using the modified Japanese Orthopedic Association (mJOA) questionnaire. It is difficult to predict which patients will recover neurological function after surgery, making it challenging for clinicians to set postoperative patient expectations. In this study, we used mJOA subscores to identify patterns of recovery and recovery timelines in patients with moderate and severe myelopathy.

**Methods:**

Fifty-three myelopathy patients were enrolled and completed the mJOA questionnaire both pre-surgery, and six weeks and six months post-surgery. Pearson chi-square tests were performed to assess relationships of both recovery patterns and recovery timelines with severity of disease.

**Results:**

Moderate myelopathy patients were significantly more likely than severe myelopathy patients to experience full recovery of upper extremity, lower extremity, and sensory domains. Disease severity did not significantly impact the timeline during which recovery occurs. Overall, >90% of patients experienced at least partial recovery by six months post surgery, 80% of which demonstrated it within the first six weeks.

**Conclusions:**

This study shows the more severe the disease experienced by myelopathy patients, the more likely they will be left with permanent disabilities despite surgery. Early identification and treatment are therefore necessary to prevent worsening quality of life and increased costs of functional dependence. The recovery timelines for each subscore are similar and provide new values to guide patient expectations in their potential post-operative recovery. The overall recovery timeline is more generalizable though potentially lacking the specificity patients seek.

## 1 Introduction

Degenerative cervical myelopathy (DCM) results in spinal cord compression and injury, often secondary to vertebral disc degeneration and herniation, hypertrophy of the ligamentum flavum, and facet arthropathy.^1^^,^^2^ Patients can exhibit a progressive loss of sensation and motor function resulting in debilitating effects on dexterity, independent locomotion, and micturition.^1^^,^^3^ Decompressive spinal surgery is the standard of care to halt the progression of symptoms and promote symptomatic improvement.^4^^,^^5^ However, the postoperative neurological recovery can be highly variable.^6^ Several tools have been designed to assess the level of impairment and track post-operative recovery in patients with DCM, including the modified Japanese Orthopedic Association (mJOA) questionnaire.^7^^,^^8^

The mJOA questionnaire is commonly used to assess the level of subjective functional impairment in DCM patients^7^^,^^8^ and is the standard for determining the severity of the disease.^9^ It consists of four questions - one for each of the major domain impairments caused by myelopathy: upper extremity motor function, lower extremity motor function, sensation, and sphincter control.^7^ Quantification of myelopathic impairment is important as it enables healthcare providers to reliably assess a patient's level of impairment and track their postoperative recovery. While the total mJOA score is used as a standard for determining the severity level of the disease and measuring overall recovery post-surgery, the pattern of neurological recovery can also be evaluated by tracking the individual domain subscores.

Given the heterogeneous nature of the disease, identifying patterns of recovery in each of the individual domain impairments (ie. upper/lower extremity motor function, sensation and sphincter control) can aid with both clinical decision-making and guiding patient expectations post-surgery. However, few studies have investigated recovery by focusing on the individual domains. Furthermore, the role that disease severity plays in recovery patterns has not yet been explored. The aim of this study is to 1) characterize the patterns of recovery in moderate and severe myelopathy patients, and 2) determine the timeline for recovery in the first six months post-surgical decompression.

## 2 Methods

### 2.1 Patient population

Patients with clinically diagnosed and imaging-confirmed DCM were enrolled into clinical and advanced imaging studies conducted by the senior investigators (N.D. and R.B.).^10^^,^^11^ The patients included in these studies met the following inclusion criteria: 1) age >18 years; 2) symptomatic progressive DCM requiring surgical intervention; 3) imaging evidence of spinal cord compression; and 4) no previous cervical spine surgery. Patients with a history of unrelated spinal cord injury, or other neurological or systemic ailments that could impair neurological function were excluded. Each subject who had agreed to participate in these studies provided written informed consent. For inclusion in this retrospective study, the patients needed to have attended a pre-operative appointment and six-week and six-month post-surgical appointments during which the mJOA was completed, and be diagnosed with moderate or severe myelopathy during the pre-operative appointment.

### 2.2 Data collection

The mJOA questionnaire was completed at the pre-operative appointment, and at the six-week and six-month post-operative appointments. The full mJOA questionnaire is shown in Table 1. Demographic data were collected at preoperative appointments.

**Table 1:** mJOA questionnaire (/18).

Category	Score	Description
UE – Upper Extremity Motor Subscore (/5)	0	Inability to move hands
	1	Inability to eat with a spoon but able to move hands
	2	Inability to button shirt but able to eat with a spoon
	3	Able to button shirt with great difficulty
	4	Able to button shirt with slight difficulty
	5	No dysfunction
LE – Lower Extremity Subscore (/7)	0	Complete loss of motor sensory function
	1	Sensory preservation without ability to move legs
	2	Able to move legs but unable to walk
	3	Able to walk on flat floor with a walking aid
	4	Able to walk up and/or down stairs with handrail
	5	Moderate to significant lack of stability but able to walk up and/or down stairs without handrail
	6	Mild lack of stability but able to walk with smooth reciprocation unaided
	7	No dysfunction
Sen. – Upper Extremity Sensory Subscore (/3)	0	Complete loss of hand sensation
	1	Severe sensory loss or pain
	2	Mild sensory loss
	3	No sensory loss
Sph. – Sphincter (Urinary Function) Subscore (/3)	0	Inability to micturate voluntarily
	1	Marked difficulty with micturition
	2	Mild to moderate difficulty with micturition
	3	Normal micturition

### 2.3 Statistical analysis

The relationship between six-month post-operative recovery and disease severity was evaluated using a Pearson chi-square test run on each mJOA subscore using pre-operative and six-month post-operative scores. Subscore values are reported as percentages or as medians ± IQR. Reporting and analysis were done independently for each subscore, and only those subjects who demonstrated impairment pre-surgery for any individual subscore were included in the analysis.

Pearson chi-square tests were also used to evaluate the relationship between disease severity and the recovery timelines of each mJOA subscore, as well as the total mJOA score. All statistical tests were carried out using SPSS v25. Statistical significance was set at an alpha of 0.05.

## 3 Results

### 3.1 Patient demographics

53 patients were included in this study, of which 23 were diagnosed with moderate myelopathy, and 30 with severe myelopathy. The severity was based on the pre-operative total mJOA score, using values that have become the standard^9^: patients were considered moderate if they had an mJOA score of 12–14, and severe if they had an mJOA score less than 12. Demographic information can be found in Table 2.

**Table 2:** Demographic data.

Characteristic	Value
Number of Patients	53
Age (years)	56.9 ± 11.7
Sex (male/female) Disease severity (moderate/severe)	38/15 23/30
Duration of symptoms (months)	17.3 ± 19.1
Side(s) affected (both/left/right/none)	44/2/4/1
MRI signal change (yes/no)	50/1
Number of levels (single/multi)	24/27
Comorbidities	
*Diabetes*	6
*Hypertension*	20
*Coronary Artery Disease*	2
*Smoker*	20

*Missing demographic data (apart from sex) for two patients.

**Fig. 1:**
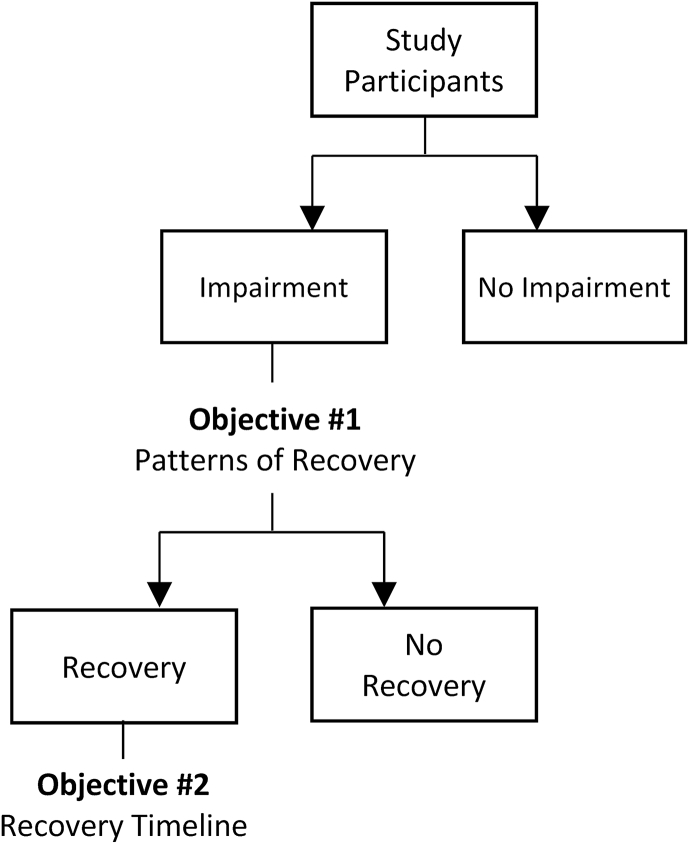
Flowchart describing how study participants are split for analysis. This process is followed separately for each domain/subscore.

The proportion of patients with pre-operative impairment in each of the mJOA domains is listed in Table 3. It is important to note that, since we are tracking the recovery process, only those patients who experienced impairment pre-surgery are included in the analysis that follows (see Fig. 1 for flowchart describing which participants are included in the various analyses.

1. Patterns of Neurological Recovery by Severity

**Table 3:** Proportion of patients experiencing impairment per-surgery in each domain.

	UE	LE	Sensory	Sphincter
Moderate (/23)	95.7 % (22)	100 % (23)	91.3 % (21)	39.1 % (9)
Severe (/30)	100 % (30)	100 % (30)	100 % (30)	50 % (15)

Values in brackets represent the number of individuals experiencing impairment. UE = upper extremity; LE = lower extremity.

**Fig. 2:**
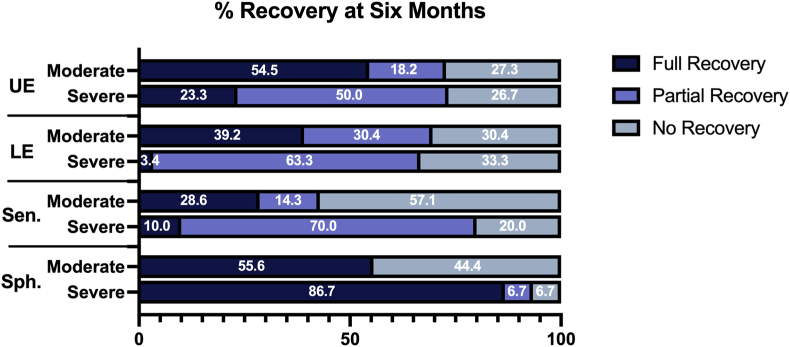
Recovery levels of moderate and severe myelopathy patients, broken down by domain. Full recovery is defined as a perfect score.

The impact of disease severity on neurological recovery at six months post-surgery is presented in Fig. 2.

The patient groups (moderate vs. severe myelopathy) were stratified into three recovery profiles: 1) those who experienced a ‘full’ recovery in an individual domain (defined as a perfect subscore), 2) those who experienced a partial recovery (defined as any recovery that did not result in a perfect subscore), and 3) those who experienced no improvement or whose impairment worsened. A Pearson chi-square test run on each subscore indicated a relationship between recovery and disease severity, except for the sphincter subscore (p-values: UE = 0.023; LE = 0.003; Sen=<0.001; Sph = 0.07). Moderate patients had a significantly greater chance than severe patients of experiencing full recovery in the upper extremity (54.5 % vs. 23.3 %), lower extremity (39.2 % vs. 3.4 %) and sensory (28.6 % vs. 10 %) subscores. A list of the median subscores at baseline and six months post-surgery are listed in Table 4.

**Table 4:** Median scores at baseline and six months.

	Moderate patients		Severe patients	
Domain (/max)	Pre-surgery	6 months	Pre-surgery	6 months
UE (/5)	4 (1)	5 (1)	2 (1)	4 (1.25)
LE (/7)	5 (2)	6 (3)	4 (1)	4.5 (2)
Sensory (/3)	2 (1)	2 (1)	1 (0)	2 (0.25)
Sphincter (/3)	3 (1)	3 (0)	2.5 (1)	3 (0)

Scores reported as medians and interquartile ranges (in brackets). UE = upper extremity; LE = lower extremity.

Also notable is the diminished recovery in the lower extremities of severe patients, as well as the attenuated recovery of sensation in both the moderate and severe patient groups.

2 Recovery timelines

Recovery timelines look at the temporal pattern of recovery post-surgery. In this study, these patterns were ascertained by categorizing patients by the follow-up visit at which they first started to display recovery (ie. six-week vs. six-month follow-up). Additionally, we identified those patients demonstrating recovery at the six-week visit who showed additional recovery at the six-month visit.

**Fig. 3:**
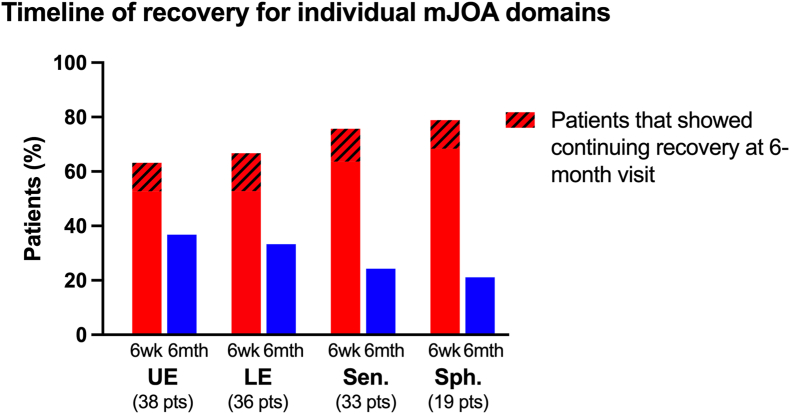
Recovery timelines for each domain, broken down by the follow-up visit at which patients first started to display recovery. The shaded part of the 6-week bar indicates the % of patients who showed additional recovery at 6 months. Sample size of each subscore listed.

A Pearson chi-square test performed on each subscore demonstrated that there was no relationship between disease severity and the recovery timeline (p-values: UE = 0.759; LE = 0.414; Sen = 0.756; Sph = 0.281). Given that there were no significant differences, as well as the small sample sizes in some of the subscores, the two severity groups were combined to produce a more generalized timeline of subscore recovery for DCM patients (Fig. 3).

**Fig. 4:**
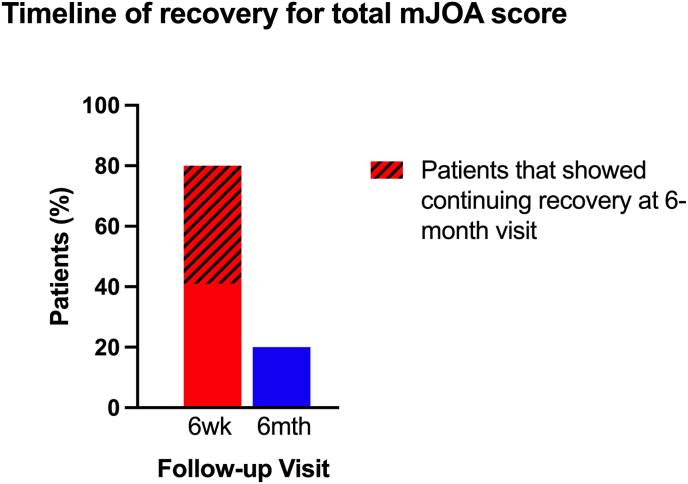
Recovery timeline for the total mJOA score, broken down by the follow-up visit at which patients first started to display recovery. The shaded part of the 6-week bar indicates the % of patients who showed additional recovery at 6 months.

As patients often display impairment in more than one domain, the recovery timeline of the total mJOA score was also investigated to give a better picture of overall recovery. A Pearson chi-square test performed on the total score demonstrated that there was no relationship between disease severity and the recovery timeline (*p* = 0.725), so the moderate and severe groups were again combined to provide a generalized timeline for overall recovery (Fig. 4). 93 % of patients (49/53) demonstrated some degree of recovery by six months post-surgery. Three moderate patients and one severe patient showed either no improvement or a small decline in their mJOA score and were not included in the analysis.

On average, 80 % of myelopathy patients experienced some form of recovery by six weeks, and approximately half that group continued to improve by six months. 20 % of patients did not show improvement in the first six weeks, but did by six months.

## 4 Discussion

In this study we sought to characterize the pattern and timeline of neurological recovery in patients with DCM and determine the influence of disease severity on surgical outcomes. This was done using the mJOA questionnaire, a patient-centred measurement that provides a total score based on subscores focusing on specific domains of impairment. This tool has become ubiquitous in defining disease severity in myelopathy patients based on the total score. In this study we provide an analysis of how severity impacts recovery in the individual subscores, in order to provide a more complete picture of the patterns of neurological recovery post-surgery.

### 4.1 Recovery patterns and disease severity

When the recovery of moderate and severe patients was compared in each mJOA subscore it was determined that disease severity played a significant role the level of recovery. A substantially higher proportion of moderate patients experienced a full recovery by six months post-surgery compared with severe patients, in the upper extremity (54.5 % vs 23.3 %), lower extremity (39.2 % vs 3.4 %) and sensory (28.6 % vs. 10 %) subscores. Essentially, the more severe the disease experienced by a patient, the more likely they will be left with permanent disabilities after surgical treatment. The importance of this observation cannot be underestimated. DCM is a progressive disease, and these results reveal that delays in diagnosis and treatment will have a significant impact on a patient's long-term independence and quality of life (QOL). QOL has ranked lower for DCM patients than many other chronic conditions, including cancer and diabetes.^12^ Delays in diagnosis are also more predictive of greater unemployment and more dependence on others,^13^ extending the impact of severe disease to the caregivers.^14^ There are also significant implications on future healthcare costs: DCM is a degenerative disease that will grow more prevalent as the population ages,^15^ and greater disability will translate to higher costs pertaining both to treatment and support services.^16^

Unfortunately, delays in diagnosis are not uncommon. Several studies have identified an average delay of 1.5–2 years before DCM was properly diagnosed.^13^^,^^17^ This is significantly longer than the six-month time-to-treatment guideline a recent meta-analysis indicated as offering a greater chance of full recovery.^18^ A recent study also concluded that duration of symptoms was an independent risk factor for post-operative efficacy, and that when the duration of symptoms was greater than 6.5 months, the prognosis was likely to be poor.^19^ The reasons for this delay are multi-faceted. The diagnostic pathway usually begins with primary care physicians, and while delayed patient presentation likely contributes, delayed detection due to the variability of clinical presentation and the lack of knowledge of cervical cord compression signs and symptoms plays a significant role.^17^^,^^20^

### 4.2 Attenuated recovery in lower extremity and sensory subscores

A diminished recovery was observed in the lower extremity subscore when compared with the upper extremity score, especially for severe patients. This may be related to the multifactorial nature of gait impairment in DCM patients.^21^ Upper motor neuron and proprioceptive dysfunction, involving multiple spinal tracts, are thought to be involved in gait impairment in DCM, although the exact mechanisms have not yet been identified.^21^ These results are also consistent with the few studies that have examined JOA subscores separately and demonstrated that recovery is greater in the upper extremities than in the lower extremities.^22^

Improvements in the sensory subscore also appeared attenuated, for both the moderate and severe patient groups. Paresthesia in the extremities is often one of the first symptoms experienced by patients^23^ and therefore one they have experienced the longest prior to treatment, making it more likely that the impairment is irreversible. However, it is also possible the results are related to the construction of the mJOA. The sensory subscore is scored 0–3 (Fig. 1), with the most common scores being ‘2’ – labeled as mild sensory loss; and ‘1’ – labeled as severe sensory loss. The coarseness of the scale means that improvements or declines in impairment, unless large, are more likely to be missed. In addition, the scores lack a clear objective definition, instead using descriptions that leave the scores open to interpretation by the testers, resulting in reduced interrater consistency.

The results of the sphincter subscore were variable, one reason being it possesses similar deficiencies to the sensory subscore, including a coarse scale and imprecise definitions for the scores. But more importantly, a high prevalence of urinary conditions unrelated to DCM occur in this patient population, making it an unreliable indicator of impairment.

### 4.3 Recovery timeline

Once a pattern of recovery was established, the second objective of the study was to determine a timeline for the recovery during the first six months post-surgery and identify any differences occurring between the domains. When investigating a timeline of recovery, the analysis was restricted to those participants who demonstrated an improvement in their mJOA scores in the first objective (Fig. 1). An initial analysis indicated that disease severity did not have an impact on how patients recovered over time in any of the domains, so the patient groups were combined to increase the sample size and improve the generalizability of the results. Focusing first on individual domains, the majority of patients experienced recovery only during the first six weeks. But since patients usually display impairment in more than one domain, this does not accurately reflect the timeline of overall recovery.

Disease severity had no impact on the rate of recovery over time for the total mJOA score, and so the patient groups were again combined. Three moderate patients and one severe patient showed no improvement, meaning that 93 % of moderate/severe patients (49/53) experienced recovery in at least one of the domains, consistent with post-surgical symptomatic improvement previously reported.^24^^,^^25^ Of those that experienced recovery, 80 % of patients improved by six weeks, half of which continued to show improvement by six months. 20 % maintained their pre-operative level of impairment at six weeks, but showed improvement by six months.

These results suggest that providing guidance to patients on what to expect during their recovery is challenging. All four domains show a similar timeline for recovery, but this is a heterogeneous patient population that usually experiences impairment in more than one domain, making it very difficult to apply these results in a practical way when advising patients on what to expect during their recovery. The overall recovery using the total mJOA score provides a better indicator, highlighting that the vast majority of patients (93 %) show some degree of recovery, and of those that do recover, 80 % will experience recovery in at least one of the domains within the first six weeks.

### 4.4 Limitations

While the mJOA is a standard tool for assessing severity in myelopathy patients, several of the domains have a coarse scale, and the assessment as a whole is not as sensitive to subtle functional changes.^26^ However, its basis in patients’ subjective experiences suggests it is a useful tool to use when determining how to guide patient expectations. Another limitation is that recovery was only monitored to six months post-surgery, but given that recovery stabilizes six months post-surgery for middle-aged patients (40–60 yrs),^27^ and the average age of patients in the study is roughly 57 years, it is unlikely that any significant changes occurring after six months were missed. Additionally, a recent study concluded that the mJOA in moderate and severe myelopathy groups demonstrated maximal improvement at three months, without further improvement at twelve months,^28^ indicating that the follow-up timeline in our study was sufficient. Finally, the smaller sample size used in this study may reduce the generalizability of the results.

## 5 Conclusions

In conclusion, this study has demonstrated that moderate patients have a greater chance at full recovery than severe patients, highlighting importance of early diagnosis and treatment. The vast majority of patients with moderate/severe myelopathy (over 90 %) will experience some degree of recovery within the first six months post-surgery, and of those patients that do experience recovery, 80 % will demonstrate it in at least one of the domains within the first six weeks post-surgery. There is no indication that different domains (ie. upper extremity motor function vs sensory) recover at significantly different rates. And finally, the mJOA questionnaire would benefit from an update that, at minimum, provides more detailed objective descriptions for scoring the sensory and sphincter subscores. This will improve the consistency of results between testers.

## CRediT authorship contribution statement

**Alexander C. Friesen:** Conceptualization, Methodology, Formal analysis, Writing - original draft. **Sarah A. Detombe:** Formal analysis, Writing - original draft, Conceptualization, Methodology. **Pat Doyle-Pettypiece:** Writing - review & editing, Investigation. **Wai Ng:** Resources. **Kevin Gurr:** Resources. **Chris Bailey:** Resources. **Parham Rasoulinejad:** Resources. **Fawaz Siddiqi:** Resources. **Robert Bartha:** Writing - review & editing, Supervision. **Neil Duggal:** Conceptualization, Supervision, Writing - review & editing.

## Declaration of competing interest

The authors declare that they have no known competing financial interests or personal relationships that could have appeared to influence the work reported in this paper.

## Abbreviations

DCM –: degenerative cervical myelopathy
mJOA –: modified Japanese Orthopedic Association
UE –: upper extremity subscore
LE –: lower extremity subscore
Sen –: Sensation subscore
Sph –: sphincter subscore
